# Isolation Hospital Expenses

**Published:** 1908-01-18

**Authors:** 


### ISOLATION HOSPITAL EXPENSES.

### DR. BARWISE ON A UNIFORM SYSTEM OF ACCOUNTS.

The Incorporated Society of Medical Officers of Health held a largely attended meeting on January 10 at 1 Upper Montague Street, Russell Square, London, when a paper was read by Dr. Sidney Barwise, medical officer for the county of Derby. Owing to the absence of Dr. George Reid, D.P.H. (President) through illness, Dr. B. C. Seaton presided.

Dr. Barwise's Lecture.

Dr. Barwise then read a paper on "Isolation Hospital Expenses."

The Discussion.

The Chairman, in inviting discussion, said the subject was one of great interest to them as medical officers. They all knew the great expense isolation hospitals necessarily entailed, and therefore the subject must prove exceedingly profitable to them all.

Dr. F. J. Burman expressed his personal indebtedness to Dr. Barwise for his paper.

Dr. Griffith Hore said this matter had claimed much attention from the Southern Society for some time. The salaries question was a most difficult one, and he had found them range from £309 to £890. This might, and had, occurred in hospitals of the same size; but the salaries were affected by the class of hospital, whether it was for a large town or a rural district. In his district they had suggested that calculations should be based upon the class of diseases admitted, the number of beds and patients, and that the average number of patients admitted should be considered. He considered in apportioning the cost of an isolation hospital that it should be based upon per thousand population. If that was a uniform basis it would be interesting to make comparisons, and it would prove a lever to show their committees that they were not spending more than was required. He had been reckoning the costs in a small hospital, and he experienced some difficulty in keeping the patients' from the staff charges. (Hear, hear.) In one case the charge for patients worked out at 7s. 0½d., and in another 3s. 8d. But he thought 7s. 6d. was too little, and yet 10s. was too much. The schedule given by Dr. Barwise, though it looked terrible, he thought was quite right.

Dr. W. Butler said the accountancy question was an important one, which Dr. Barwise's schedule should go far to solve. He found that clerks were very liable to place the figures in the wrong column.

Dr. H. Meredith Richards said in his hospital they had adopted a modified Burdett system. They found a difference in the cost of beds of as much as 30 to 40 per cent. in a year; but this was due to the cases treated, there being a great difference in the cost of treatment between scarlet fever and diphtheria. Dr. Barwise's idea of dividing the staff from patients' expenses was, to his mind, a wise one, for if a division was made, where the cost of the patient ran up so high the proportion of money spent on the staff would not be so comparatively large. The idea of the Local Government Board to make the dietary of a fixed character and sufficiently large for a strong healthy person was absurd, and for the auditor to compare the provisions ordered with the scale laid down could only lead to such incidents as occurred at Hoxton Asylum, where the sugar was poured down the drains—or the officials' private larders must be overstocked so as to show there were not too much goods in. A good method was to get the figures of the provisions and food supplied in monthly quantities where there was a suspicion that waste occurred, and compare them with the corresponding period of the previous year. He thought Dr. Barwise would have accomplished a useful work if he could succeed in instituting a uniform system of accounts.

Dr. D. S. Davies (Bristol) said with a uniform system of accounts the cost per head must necessarily be higher in a small hospital than in a large one.

Dr. Herbert Jones said in rural districts the hospital accounts were kept in the same way as the workhouse accounts. The staff and patients were put upon rations. In Herefordshire they had two hospitals, one with four beds and one with nine. He had charge of the one with nine beds. The smaller one was worked on the rations principle, and what happened was that the rations not used were carefully placed on one side and someone's larder was greatly increased. In his nine-bed hospital no rations were served. The principle adopted was that the nurse in charge was allowed to order what was required, and (say at the end of a period of six weeks) he went through the housekeeping accounts, and having reckoned up the number of patients treated and the number of days they occupied beds, he worked out the cost. If that cost worked out at more than 6s. per week he closely scrutinised the bills, but he must say that rarely did it exceed 5s. 6d. per week. This was about 20 per cent. lower than at the hospital where rations were given. Yet when the attention of that hospital's committee was drawn to the fact, they replied that there could be no waste for fixed rations only were allowed.

Dr. F. E. Freemantle said he made an exhaustive study of the question a few years ago, but he could obtain no figures of value. He hoped as an outcome of the paper that a uniform system of accounts would be adopted, and so help to economise in these hospital expenses, which were always much grudged. He would suggest that the paper be referred to the Council of the Society with a view to their asking the Local Government Board to adopt a uniform system of dietary and accounts.

Dr. Barwise, replying upon the discussion, accepted the suggestion of the last speaker, and further suggested that in Sub-section 3, Section 17 of the Act, that the words "Local Government Board" should be substituted for "Hospital Committee." He quite agreed that in the statements which should be prepared the character of the disease should be stated, together with surrounding facts as to each case. Upon the dietary point he had discussed the matter with the Local Government Board auditor, and he believed something would be fixed on the lines he had suggested.

It was then decided to refer the paper to the Council as suggested, and to ask the Local Government Board to amend Section 17 as indicated.

